# Developmental Coordination Disorder in Chinese Children Is Correlated With Cognitive Deficits

**DOI:** 10.3389/fpsyt.2019.00404

**Published:** 2019-06-13

**Authors:** Li Ke, Wen Duan, Ye Xue, Yun Wang

**Affiliations:** ^1^State Key Laboratory of Cognitive Neuroscience and Learning, Beijing Normal University, Beijing, China; ^2^Collaborative Innovation Center of Assessment for Basic Education Quality, Beijing, China

**Keywords:** developmental coordination disorder, movement development, cognitive ability, cognitive deficits, execution function

## Abstract

Cognitive deficits have been commonly observed in children with developmental coordination disorder (DCD), including memory, attention, and executive function difficulties. The present study evaluates the specific cognitive deficits in Chinese children with DCD, through a number of tests. A total of 401 children aged 7 to 10 years old from primary schools in Guangdong Province, China, participated in this study. Using the guidelines of the *Movement Assessment Battery for Children* (“Movement ABC-2”), a measurement tool of motor function ability, the children were divided into a DCD group, a group identified as being at risk of DCD, and a normal control group. The results of our analysis revealed that children’s overall motor abilities could predict their overall cognitive ability, reaction time, memory, and attention. The performance of the DCD children was worse than that of the other two groups in terms of reaction time. The DCD group also returned lower scores for executive function than the normal control group did. A regression analysis showed that the cognitive deficits in children with DCD center mainly on poor executive function rather than attention and memory issues. These findings provide preliminary results regarding the cognitive deficits in Chinese children with DCD and have potential applications for the diagnosis and treatment of the disorder.

## Introduction

Movement development corresponds to other basic abilities and has attracted much attention from researchers as well as health-care providers. Previous studies have labeled children’s movement difficulties as clumsiness, dyspraxia, motor learning difficulties, or as a disorder of attention, motor control, and perception (“DAMP”). These concepts explain children’s problems with movement or the acquisition of movement from different perspectives (1–5). However, such terminologies only focus on a particular field or the children’s symptoms but not their developmental process (6). Through in-depth explorations, researchers have ultimately reached a consensus that the developmental coordination disorder (DCD) of children should be classified as a subcategory of a neurodevelopmental disorder (7, 8).

Currently, DCD is generally identified as an impairment of motor ability distinct from intelligence and other neurological disorders (8). With the development of brain and cognitive neuroscience research, though, it has been discovered that DCD is not just relevant to the movement system, but is also related to a complex series of neurological dysfunctions involving memory, attention, executive function, and reasoning (9, 10), each of which is discussed further in the paragraphs that follow. Additionally, neuroimaging data has shown altered brain activation patterns across functional networks involving prefrontal, parietal, and cerebellar regions in children with DCD, compared to children in other groups (11–13), which overlap with a couple of key structures within the internal modeling regions (14). Moreover, researchers have found evidence that is suggestive of a relationship between DCD and profound cognitive deficits. Research in overweight 7-11 year-old children found that exercise improved executive function and altered brain activation (15).

Children with DCD often suffer a loss of visual–spatial memory, which is significantly correlated with the planning and control of their movements (16, 17). This may be due to a different number of cognitive resources being allocated to visual–spatial memory during the process of executing actions (18). Furthermore, a close relationship between visual–spatial memory and learning ability among children with DCD (19) has been found, with research showing that, among children with DCD, those with lower visual–spatial memory perform less well in tests on their literacy and computation abilities (20). The particular correlation between visual–spatial memory and learning ability indicates that the processing of working memory and visual–spatial memory plays an important role in the acquisition process of movement development for children with DCD.

Furthermore, children with DCD also exhibit poor performances in attentional tasks that require autonomous processing of visual–spatial information, such as cue information (21), visual tracking (22), and simultaneous responses to dual tasks (23). Prior research featured direction-hiding visual–spatial attention tasks to verify the relationship between DCD and a lack of selective attention, and found that children with DCD experienced longer reaction times in the presence of external disturbances, in comparison with children in the normal control group (24, 25). Subsequently, Wilmut et al. (26) found that the slow response to exogenous attention distribution among children with DCD was only observed in tasks involving the integration of visual actions. To further clarify the relationship between attention and movement performance among children with DCD, more recent studies have used electroencephalogram recordings taken from the foreheads of participants and subsequently converting them into indicators of attentional levels, with the results indicating a correlation between attention level and movement performance among children with DCD (27).

“Reaction time” represents an individual’s fastest response to the occurrence of a stimulus. Researchers have found that the cognitive decision-making processes associated with responding to choices results in the relatively slower movements of children with DCD; that is, they have longer reaction times than other children (21, 28–30). These findings suggest that children with DCD have difficulties processing movement information.

Dysfunction in execution is also an important characteristic of children with DCD. Indeed, a meta-analysis of research, carried out during the period of 1997 to 2011, and relevant to the assessment of movement planning, inhibition, working memory, and cognitive flexibility indicated significant executive dysfunction (31). “Executive function” is a complex process comprising higher-order cognitive thought and action that involves working memory, inhibition control, and cognitive flexibility (32). Impaired executive function can impact metacognition when making movement plans, which in turn suggests that children with DCD may experience extensive problems with regard to this aspect too.

Our analysis of previous research establishes that cognitive deficits are commonly seen in children with DCD, as deficits can be observed with respect to their memory, attention, and executive function. These prior findings prompted our current research question, as follows: What is the correlation between memory, attention, and executive function among children with DCD—and is there a relationship of inclusion between these factors? As previous studies have mainly featured Western children as participants, we posited a further question of whether similar results could be obtained in China. For the present study, we screened a designated sample size of children for DCD using a standardized tool with a credible norm. We subsequently conducted cognitive tests, involving memory, attention, executive function, and reaction time. The purpose of our research was to verify and analyze the overall impact of DCD on children’s cognitive abilities and specific cognitive functions, such as memory, attention, and executive function, as well as to explore cognitive deficits in the dynamic mechanisms underlying children’s movement development and difficulties.

## Materials and Methods

### Participants

A total of 401 children from three primary schools in Guangdong Province, China, participated in the movement and cognitive tests in this study. The children were aged 7 to 10 years old (mean age = 8.98 ± 1.10 years) and the sample consisted of 214 boys and 187 girls. The study protocol was screened and approved by the Institutional Review Board of the National Key Laboratory of Cognitive Neuroscience and Learning, Beijing Normal University. Parents were provided with an explanation of the study’s procedures. Written and informed consent were obtained from the parents. After the deletion of missing data, the final sample comprised 399 participants. The gender and age distributions of all tested children are shown in **Table 1**.

**Table 1:** Age and gender distributions of participants.

Age	Gender	No. of Children	%
7 years old	Boys	37	64.9
	Girls	20	35.1
8 years old	Boys	45	60.8
	Girls	29	39.2
9 years old	Boys	40	55.6
	Girls	50	48.9
10 years old	Boys	92	64.9
	Girls	88	35.1
Total		401	

Using the guidelines of the *Movement Assessment Battery for Children* (second edition, also known as “Movement ABC-2”), a measurement tool of motor function ability, children with a total score of 5 points or fewer were assigned to the DCD group; children with a score of 6 were identified as being “at risk” of DCD, and assigned to the AR group; and children with a score of 7 or more were identified as “normal controls” and placed in the NC group accordingly. Applying this standard, the resultant DCD group comprised 75 participants, the AR group had 35, and the NC group featured 291 participants.

### Methods

#### Movement Assessment Battery for Children

The children completed the Movement ABC-2’s “age band 2” tests for 7- to 10-year-old (6). Each age band encompasses three dimensions: 1) manual dexterity, comprising three items; 2) aiming and catching, featuring two items; and 3) balance, with three items. For children aged 7 to 10 years, a “normal” model was provided for the span of every single year. Through comparison with these normal models, a standard conversion was used for the tasks, dimensions, and total scores. The converted standard score was 10, the standard deviation was 3, and the scores ranged between 1 and 19.

In 2016, the Chinese norm for children aged between 3 and 10 years was established using this tool. Through stratified sampling, all of China was divided into seven regions: North China, East China, South China, Central China, Southwest China, Northeast China, and Northwest China. Based on the proportion of children under 10 years old in the 2010 census data, the regional sample size was distributed, and data collected from 2,185 children, incorporating 1,143 boys (52.3%) and 1,042 girls (47.7%), showing reasonable gender distributions at all ages. Following data cleaning, analysis, and standard demarcation, the converted standard scores of the Chinese norm were obtained. The test–retest reliability of this Chinese norm is 0.86 and 0.859 in intraclass correlations. The results of confirmatory factor analyses show that the comparative fit index is 0.937, the root mean square error of approximation is 0.045, and the Tucker–Lewis index is 0.897, signifying good construct validity (33).

#### Measurement of Cognitive Abilities

Four core abilities were measured: 1) attention, 2) memory, 3) reaction time, and 4) execution function. Selective attention and continuous attention tasks were used to measure attention. Associative memory and short-term memory tasks were chosen to evaluate memory. Processing speed tasks were used to test reaction time, and impulse control and information-updating tasks were used to measure execution function. The children were asked to complete all cognitive tasks independently on a computer within 30 min. All scores were added, and the total scores were transformed into a standard score with a mean of 100 and a standard deviation of 15.

#### Family Background Information

The parents of participants were asked to fill out a questionnaire in order to provide information regarding each child’s mother’s years of education, father’s years of education, and annual household income. These variables were controlled during the data analysis.

#### Measurement of Movement Development

Prior to these measurements being taken, an email was sent to the school administrators that included the Institutional Review Board approval letter, the task lists, and informed consent. These were then distributed to the students’ parents or caregivers. The online testing system generated lists of identification numbers for each school according to the number of students who volunteered to complete the study (via their parents). The data were collected *via* a face-to-face test using the Movement ABC-2 measurement tool on all the participating children.

#### Measurement of Cognitive Ability

Children completed their cognitive assessment on a school computer. The interval between the measurement of movement development and the measurement of cognitive ability was more than 2 h.

#### Data Analysis

SPSS 20.0 was used for the analyses of the Pearson correlations, multiple linear regression, and multivariate covariance analyses.

## Results

### Correlations Between Movement Development and Cognitive Abilities

The correlations between the total scores for the children’s movement development (all dimensions) and cognitive abilities were calculated respectively, and the results are presented in **Table 2**. As can be seen, the total scores of the children’s movement development and their total cognitive scores are significantly correlated (*r* = 0.18, *p* < 0.01), and movement development is also highly correlated to reaction time (*r* = 0.14, *p* < 0.01), memory (*r* = 0.14, *p* < 0.01), attention (*r* = 0.10, *p* < 0.05), and executive function (*r* = 0, *p* < 0.05). The manual dexterity dimension of movement development is significantly correlated to memory (*r* = 0.13, *p* < 0.05), executive function (*r* = 0.11, *p* < 0.05), and the total cognitive scores (*r* = 0.12, *p* < 0.05). The aiming and catching dimension are closely related to attention (*r* = 0.14, *p* < 0.01) and total cognitive score (*r* = 0.14, *p* < 0.01). The balance dimension of movement development is largely correlated to reaction time (*r* = 0.17, *p* < 0.01) as well as total cognitive score (*r* = 0.13, *p* < 0.05).

**Table 2:** Correlations between cognitive development and movement development.

		1	2	3	4	5	6	7	8	9
1	Cognition—reactive time	1								
2	Cognition—memory	0.34**	1							
3	Cognition—attention	0.21**	0.20**	1						
4	Cognition—executive function	0.17**	0.12*	0.33**	1					
5	Total cognitive score	0.54**	0.47**	0.76**	0.59**	1				
6	Movement—manual dexterity	0.08	0.13*	0.05	0.11*	0.12*	1			
7	Movement—aiming and catching	0.05	0.09	0.14**	0.07	0.14**	0.20**	1		
8	Movement—balance	0.17**	0.08	0.04	0.08	0.13*	0.37**	0.18**	1	
9	Total movement development score	0.14**	0.14**	0.10*	0.12*	0.18**	0.71**	0.57**	0.82**	1

*p < 0.05.

**p < 0.01.

### Relationship Between Movement Development and Cognitive Development

For the multiple linear regression analysis, the total score for movement development was used as the independent variable; the total score for cognitive abilities as the dependent variable; and the children’s genders, ages, parental years of education, and annual family income as the control variables. The results are shown in **Table 3**, where it can be observed that overall motor ability has a significant predictive effect on the overall cognitive ability of the children (β = 0.22, *p* < 0.001).

**Table 3:** Predictive effect of total movement development score on total cognitive score.

	*β*	*t*	Δ*R* ^2^	Δ*F*
Gender	−0.09	−1.56		
Age	−0.07	−1.19		
Mother’s years of education	−0.08	−1.10	0.02	1.08
Father’s years of education	0.06	0.89		
Annual household income	−0.01	−0.22		
Total movement development score	0.22***	3.89	0.05***	15.11

***p < 0.001.

The individual scores for reaction time, memory, attention, and executive function were then used as dependent variables for further multiple linear regression analysis, with the independent and control variables as above. The results are presented in **Tables 4** to **7**, wherein the overall motor abilities of the children show a significant predictive effect on their reactive abilities (β = 0.19, *p* < 0.001), memory (β = 0.20, *p* < 0.001), and attention (β = 0.14, *p* < 0.05), whereas it shows little predictive effect on the children’s executive function (β = 0.10, *p* > 0.05).

**Table 4:** Predictive effect of total movement development score on reaction time score.

	*β*	*t*	Δ*R* ^2^	Δ*F*
Gender	−0.01	−0.23		
Age	0.33***	5.98		
Mother’s years of education	−0.09	−1.32	0.11***	7.59
Father’s years of education	0.08	1.13		
Annual household income	0.00	0.02		
Total movement development score	0.19***	3.42	0.03**	11.72

**p < 0.01.

***p < 0.001.

**Table 5:** Predictive effect of total movement development score on memory score.

	*β*	*t*	Δ*R* ^2^	Δ*F*
Gender	−0.09	−1.58		
Age	0.13*	2.22		
Mother’s years of education	−0.15*	−2.16	0.04*	2.29
Father’s years of education	0.04	0.55		
Annual household income	−0.02	−0.43		
Total movement development score	t0.20**	3.47	0.04**	12.06

*p < 0.05.

**p < 0.01.

**Table 6:** Predictive effect of total movement development score on attention score.

	*β*	*t*	Δ*R* ^2^	Δ*F*
Gender	−0.12*	−2.10		
Age	−0.32***	−5.90		
Mother’s years of education	−0.10	−1.57	0.13***	8.86
Father’s years of education	0.06	0.96		
Annual household income	0.00	−0.08		
Total movement development score	0.14*	2.51	0.02*	6.31

*p < 0.05.

***p < 0.001.

**Table 7:** Predictive effect of total movement development score on executive function score.

	*β*	*t*	Δ*R* ^2^	Δ*F*
Gender	0.01	0.16		
Age	−0.18**	−3.09		
Mother’s years of education	0.08	1.16	0.04*	2.30
Father’s years of education	−0.09	−1.24		
Annual household income	0.01	0.16		
Total movement development score	0.10	1.65	0.01	2.73

*p < 0.05.

**p < 0.01.

### Cognitive Abilities of Children With DCD

In order to study the cognitive differences between the DCD group, AR group, and NC group, a multivariate analysis of covariance was conducted with the three groups as independent variables; cognitive ability, reactivity, memory, attention, and executive function scores as dependent variables; and the children’s genders, ages, parental years of education, and annual family income as covariates. The results are shown in **Table 8**.

**Table 8:** Comparison of the scores of the DCD group, AR group, and NC group in all dimensions.

Group	Cognitive Score *M* (*SD*)	Reactivity *M* (*SD*)	Memory *M* (*SD*)	Attention *M* (*SD*)	Executive Function *M* (*SD*)
DCD group (1)	89.09 (7.31)	81.17 (11.61)	96.98 (6.59)	80.45 (19.50)	89.47 (12.31)
AR group (2)	91.66 (8.36)	89.92 (10.40)	97.88 (5.87)	80.84 (20.08)	90.72 (10.18)
NC group (3)	92.97 (8.37)	86.54 (11.77)	98.89 (8.12)	86.26 (20.16)	93.92 (12.80)
*F* value	5.65**	5.59**	1.75	2.93	3.28*
partial Eta-Squared	0.04	0.04	0.01	0.02	0.02
Post hoc test	1 < 3**	1 < 2**, 1 < 3**			1 < 3*

*p < 0.05.

**p < 0.01.

Here, there are significant differences in the reactivity score, *F*(2, 396) = 5.59, *p* < 0.01; executive function score, *F*(2, 396) = 3.28, *p* < 0.05; and total cognitive score, *F*(2, 396) = 5.65, *p* < 0.01. We conducted a *post hoc* test using the LMATRIX syntax in SPSS, and the results verify that the performance of the DCD group was worse than that of the NC and AR groups in terms of reactivity. With regard to executive function and cognition, the DCD children scored significantly lower than the NC group’s children.

## Discussion

Movement development has an observable influence on children’s basic cognitive abilities. In this study, a standardized movement test (featuring 2016 Chinese norm data) and various cognitive ability tasks were used to test the movement development levels and cognitive abilities of children aged 7 to 10 years. The results suggest that there is a significant correlation between children’s movement development levels and the total scores from the cognitive tests, a finding that is consistent with the relationships identified between the children’s movement development levels and individual cognitive tests. Specifically, the total score for movement development is largely correlated with the scores of reaction time, executive function, memory, and attention tasks. These results are consistent with the findings and general direction of existing studies, corroborating that children’s movement development may have an impact on their memory, attention, executive function, and other cognitive abilities.

The cognitive ability deviation in children with DCD manifests mainly with respect to executive function. After distinguishing between the DCD, AR, and NC group according to the norms, the results of our study’s multivariate covariance analysis showed that DCD children scored significantly lower in terms of the total cognitive score compared to the other groups, which further confirmed that children with DCD generally have deviations in cognitive ability. In terms of specific cognitive tasks, this study found that, compared with the other groups, children with DCD attained significantly lower reaction time and executive function scores, which is consistent with the findings of previous studies. The main cognitive tasks that tested the children’s reaction time and executive function in this study reflected the three principal components of executive function (cognitive flexibility, inhibition control, and working memory), and the results support the conclusion of previous studies that children with DCD have difficulty with executive function (31, 34).

On the other hand, this study found that children in the DCD, AR, and NC groups did not show significant differences in cognitive task performance with regards to attention and memory, which differs from the conclusions of previous studies. We believe that performance deviations in respect of attention for children with DCD in existing research, occur mostly in visual tracking, due to the interference of invalid clues and other tasks (24, 25). Such tasks are performed through inhibition control, a principal component of executive function. Other studies have pointed out that attention performance deviation in children with DCD occurs in dual-task parallel switching (23), and attention switching—or cognitive flexibility—is also a principal component of executive function. The results in the current study, pertaining to the reaction time scores (cognitive flexibility) and executive function scores, are consistent with those of prior studies. However, regarding other cognitive tasks that examined attention only, including attention maintenance, there was no significant difference between the DCD, AR, and NC group.

Similarly, there have been some reports on performance deviation regarding memory in children with DCD that also point to the task paradigm related to children’s visual perception of spatial information and the retention of motor information (18, 35, 36)—a task paradigm that involves working memory, the third principal component of executive function. The results of executive function tasks in our study support this conclusion regarding working memory, but, when carrying out other memory tasks, there was no significant difference in the performance of children with DCD in terms of the total memory score. These findings lead us to conclude that the deviations in attention and memory in children with DCD reported in previous studies were, rather, deviations in those components of executive function associated with attention switching and working memory. The cognitive bias in children with DCD centers mainly on poor executive function rather than deficits in attention or memory.

Our study has selected and systematically discussed the cognitive abilities most affected by DCD as proposed by previous studies. As well as verifying the results of prior research, we inferred further that the influence of DCD on cognitive ability is mainly reflected in a child’s executive function. However, due to funding and time restrictions, the present study was unable to select a larger number of more nationally representative children with DCD as samples, or to obtain a wider distribution in age/school grade, and subsequently to discuss possible regional and age differences. Another limitation of this study was the selection of the cognitive task paradigm. In order to save on costs and time, in the children’s individual tests, we chose to administer a computer-aided cognitive test rather than a cognitive task paradigm based on a range of surveys in the laboratory. This created an objective restriction that prevented further discussion of the effect of DCD on cognitive ability. Finally, an additional limitation of this study concerns the cross-sectional analysis, which limited us in terms of being able to perceive the development trends. This issue will be addressed in future research.

## Data Availability Statement

The datasets generated for this study are available on request to the corresponding author.

## Ethics Statement

Approved by IRB of Beijing Normal University.

## Author Contributions

LK co-designed the study, collected the data, conducted most of the analyses, and wrote the first draft of the manuscript. YW co-designed the study and contributed to the writing and revising of the first draft. WD provided training for the experimenters and collected the data. YX helped in the analyses of the results and edited the language.

## Funding

This study was supported by a grant from the Fundamental Research Funds for the Central Universities (grant number: ZYGX2015J167).

## Conflict of Interest Statement

The authors declare that the research was conducted in the absence of any commercial or financial relationships that could be construed as a potential conflict of interest.
